# Dynamic compression of Ce and Pr with millisecond time-resolved X-ray diffraction

**DOI:** 10.1038/s41598-022-22111-5

**Published:** 2022-10-14

**Authors:** Earl F. O’Bannon III, Rachel J. Husband, Bruce J. Baer, Magnus J. Lipp, Hanns-Peter Liermann, William J. Evans, Zsolt Jenei

**Affiliations:** 1 Physics Division, Physical & Life Sciences Directorate, Lawrence Livermore National Laboratory, Livermore, CA 94551 USA; 2 Deutsches Elektronen-Synchrotron DESY, Notkestr. 85, 22607 Hamburg, Germany

**Keywords:** Phase transitions and critical phenomena, Phase transitions and critical phenomena, Structure of solids and liquids, Metals and alloys

## Abstract

Both cerium (Ce) and praseodymium (Pr) undergo a volume collapse transition under compression that originate from similar electronic mechanisms. Yet the outcome could not be more different. In the case of Ce with one affected 4f electron the volume collapse leaves the crystal symmetry intact, whereas for Pr with two 4f electrons the crystal symmetry changes from a distorted face centered cubic structure to a lower symmetry orthorhombic structure. In this paper, we present a study of the effect of strain/compression rate spanning nearly 4 orders of magnitude on the volume collapse phase transitions in Ce and Pr. These dynamic compression experiments in a diamond anvil cell also reveal kinetic differences between the phase transformations observed in these two materials. The transition cannot be overdriven in pressure in Ce, which indicates a fast kinetic process, whereas fast compression rates in Pr lead to a shift of the phase boundary to higher pressures, pointing to slower kinetics possibly due to the realization of a new crystal structure.

## Introduction

One of the fundamental difficulties associated with shock compression experiments is the inherent entanglement of the pressure and temperature of the final state. Shock compression to a given stress (“pressure”) results in a change in density but also an increase in internal energy greater than the isentropic compression work to the same density as shown by the Rankine–Hugoniot equations^1^. This additional energy leads invariably to a concomitant large temperature increase^2^ which can be large enough to be measured using pyrometry techniques^3,4^. Experimental uncertainties in temperature measurements can be in the hundreds or thousands of Kelvin due to low photon counts at such short (ns to µs) timescales. In addition, there are other (unknown) influences like thermal diffusivity and conductivity of windows^5^ that make an accurate determination of temperature very challenging. Large scale experimental campaigns have been undertaken or are still ongoing to develop temperature diagnostics^6^. This led to the development of graded density impactors for light-gas guns^7^ and nearly isentropic dynamic compression techniques^8–11^ which minimize sample heating by performing ‘shockless’ compression. In addition, the short timescales of dynamic compression experiments may result in non-equilibrium material behavior^2,12^.

While shock compression studies necessarily approach the experimental determination of the ambient temperature equation of state (EOS) from the (very) high temperature regime, there are other techniques that are more suitable for the determination of this part of the EOS. Static compression in large volume presses and diamond anvil cells (DACs) does not result in an increase in the sample temperature because the pressure increase is very slow. Heat generated due to work done on the sample (PdV, where P = pressure and dV = the change in volume) or the generation of dislocations and other defects can therefore be dissipated in these experiments. Dynamic DACs (dDACs) can access compression rates between conventional dynamic and static compression techniques^13,14^. However, the question arises whether such fast compression rates in a dDAC would result in an increase in the sample temperature as compression timescales could be too short to allow for heat to fully dissipate. This, in turn, could result in a shift of the phase transition pressure to higher or lower pressures, depending on the P–T slope of the phase boundary. However, the extremely high thermal conductivity of the diamond anvils (2000 W/mK at ambient pressure) means that samples that are in contact with the diamond cool on μs timescales^15^, and so the possibility of sample heating has typically been ignored in previous dDAC experiments^16^.

The dynamic compression behavior of the lanthanides cerium (Ce) and praseodymium (Pr), which have one and two 4f electrons, respectively, were investigated in this study. Both materials exhibit a large volume collapse (VC) transition driven by the behavior of the 4f electrons^17–24^, where the fractional volume change of the VC transition is very sensitive to temperature. Ce adopts the face centered cubic (*fcc*) γ-Ce structure at ambient conditions and undergoes an isostructural transition to the fcc α-Ce structure at ~ 0.7 GPa on compression. The γ–α transition is associated with a ~ 15% volume reduction at room temperature, and the phase boundary has a positive *P–T* slope with a gradient of ~ 250 K/GPa, terminating in a critical point at ~ 480 K^21^. A similar decrease in the volume change of the VC transition takes place in Pr^20^. At room temperature there is a ~ 10% decrease in volume across the VC transition and the phase boundary has a positive *P–T* slope with a gradient of ~ 100 K/GPa. However, the similarities end there, while Ce’s phases are isostructural on both sides of the transition (*fcc*), Pr’s are distinctly different. At ambient pressure Pr adopts the double hexagonal close packed (dhcp) structure (Pr-I) and above ~5.2 GPa it transforms to an *fcc* structure (Pr-II). At ~7-8 GPa Pr transforms to a distorted face centered cubic (*d-fcc*) structure (Pr-III) and at ~20 GPa Pr collapses into a lower-symmetry α-uranium (α-U) structure (Pr-IV). The bulk modulus of Ce under compression decreases until it reaches a minimum and then begins to increase as pressure increases^21^. Conversely, the bulk modulus of Pr behaves “normally”, under compression by increasing and a discontinuous increase in the bulk modulus is observed across the VC transition^25^. While both Ce and Pr have been extensively studied under static compression conditions^20–22,25–32^, far fewer dynamic compression studies have been reported^33–35^, and to-date there have been no studies at compression rates that are in-between static and dynamic shock or ramp compression regimes.

## Results and discussion

**Figure 1:**
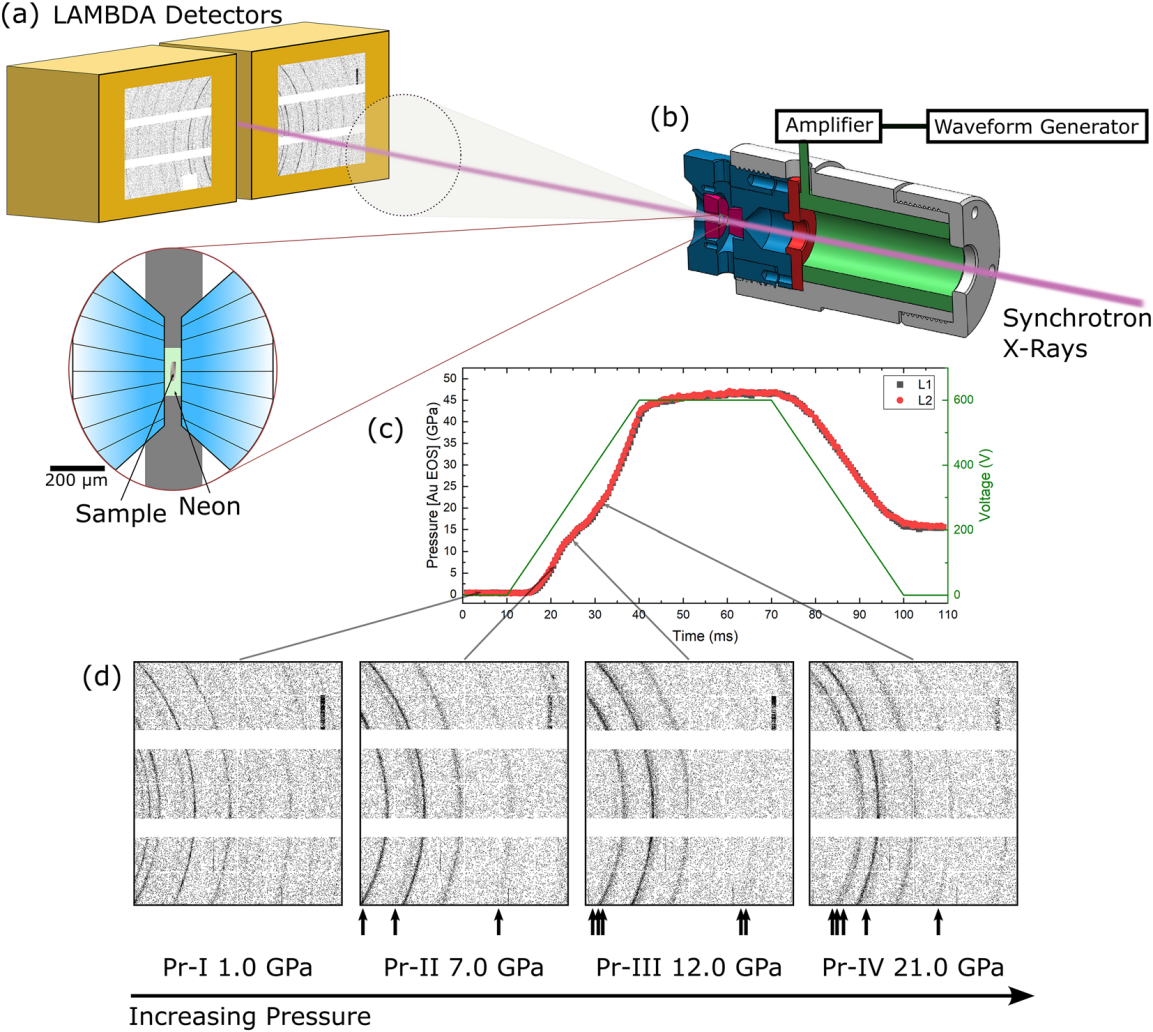
Dynamic DAC experimental setup with fast 2D X-ray diffraction at the extreme conditions beamline P02.2 at PETRA III. (**a**) Two GaAs LAMBDA photon counting X-ray detectors are positioned on either side of the X-ray beam, where each detector collects a portion of the diffracted Debye–Scherrer rings. (**b**) Schematic of the piezo-driven dynamic DAC showing how the X-rays pass through the middle of the piezo actuator to interact with the sample that is contained in a gasket between the two diamond anvils (inset zoomed into the sample compartment). (**c**) Typical pressure–time profile obtained from a ramp compression experiment with the drive voltage is also shown. (**d**) 2D raw diffraction patterns from four solid phases of Pr obtained on the compression portion of the ramp, where the most intense reflections from the high-pressure Pr phases are indicated with the arrows.

A total of eight compression ramps on Ce and seventeen ramps on Pr samples were carried out at the Extreme Conditions Beamline (ECB, P02.2) at PETRA III. Figure 1 shows a schematic of the experimental set-up for a typical dDAC experiment, where X-ray diffraction data are collected using two GaAs LAMBDA detectors positioned at opposite sides of the incoming X-ray beam. Samples were compressed with average compression rates ranging from ~ 10 to > 1000 GPa/s, and data were collected with acquisition times ≥ 0.5 ms. More than 20,000 diffraction patterns were collected during this study, which allowed us to characterize the structural evolution of Ce and Pr across the entire pressure range of each dDAC ramp at various compression rates.

**Figure 2:**
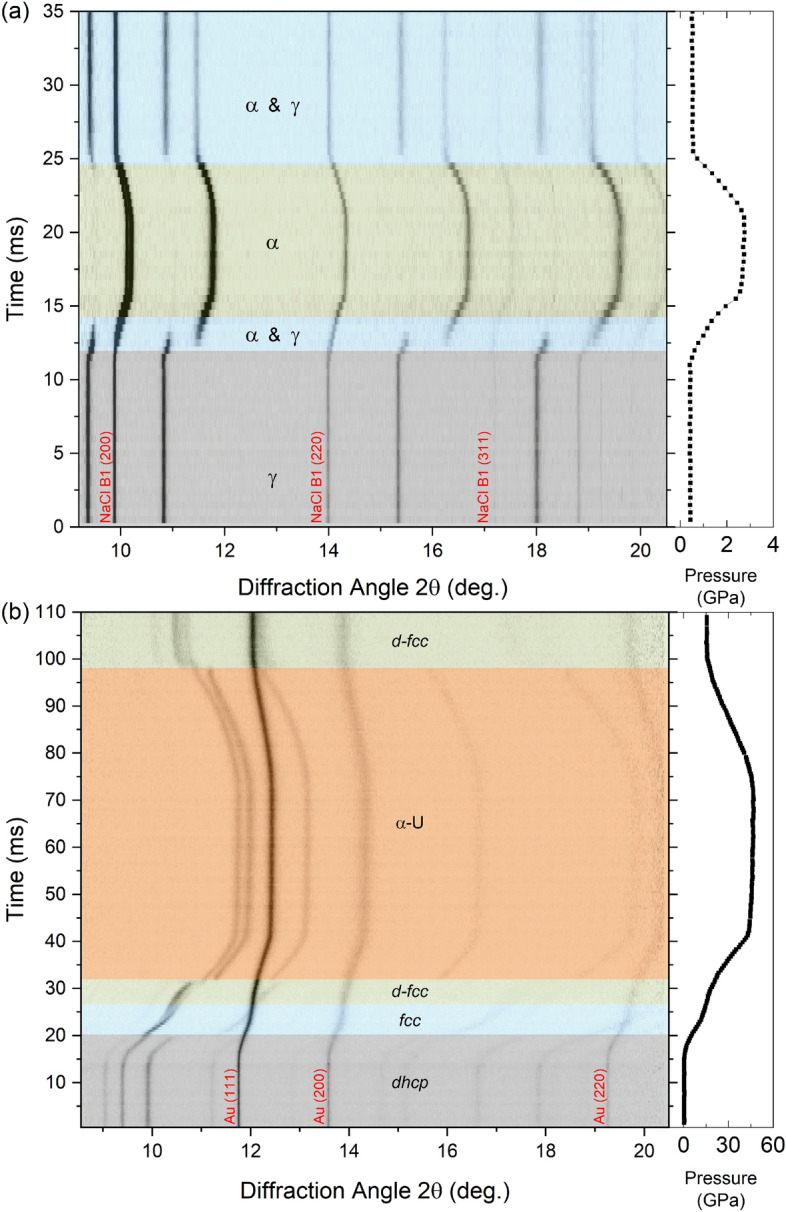
Representative dynamic compression intensity plots of (**a**) Ce compressed at an average compression rate of 472 GPa/s and (**b**) Pr at an average compression rate of 1547 GPa/s. In Ce, the γ-α VC transition is clearly observed, which is identified by the discontinuous shift of the *fcc* reflections to higher 2θ at ~ 12 ms. For Pr the entire structural sequence is observed in this run: the sample transforms from the ambient pressure *dhcp* Pr-I structure to *fcc* Pr-II at ~ 20 ms followed by a transition to *d*-*fcc* Pr-III at ~ 26 ms and finally into the collapsed α-U Pr-IV phase at ~ 31 ms. Side panels on each plot show the pressure profile for each experiment.

Representative intensity plots from Ce and Pr dDAC experiments are shown in Fig. 2. A complete list of all experimental runs can be found in the Supplementary Material. High-pressure phase transitions in Ce and Pr are easily identified in these intensity plots by the appearance of new diffraction peaks, disappearance of peaks, or the discontinuous shifts of peaks. While, reflections from the pressure marker appear as continuous lines that shift smoothly to higher diffraction angles during compression and to lower diffraction angles on decompression. In all cases, the sample pressure did not return to its initial value on decompression, which is typical in these experiments due to the nature of the gasket deformation during loading and unloading process. This was not typically an issue in the Pr experiments, as the sample always back-transformed to Pr-III on decompression. However, due to the relatively large hysteresis of the VC transition in Ce, either pure α-Ce or a mixed α/γ phase was observed at the end of each ramp. Therefore, the pressure of the Ce samples was manually lowered to ≤ 0.2 GPa after each compression cycle so that the sample would transform back to γ-Ce before the start of the next ramp. This was achieved by loosening the dDAC cap that holds the piezo actuator against the DAC and releasing the screws on the DAC.

**Figure 3:**
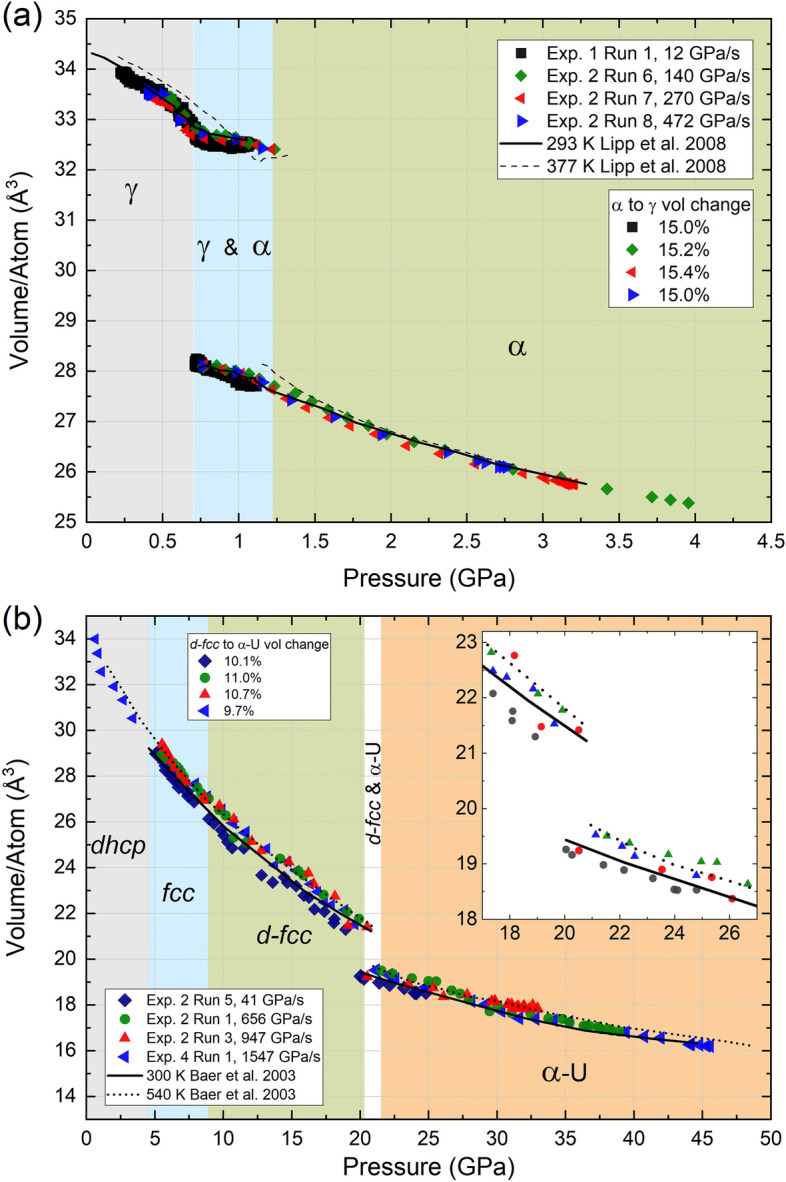
Representative *P*–*V* curves collected at various compression rates for (**a**) Ce and (**b**) Pr. Multiple runs at different compression rates are plotted to highlight that there are no systematic changes in the P–V curves collected at different compression rates. Additionally, elevated temperature isotherms are plotted in both panels to assess whether any significant heating of the sample occurs under rapid compression at room temperature in the dDAC.

The pressure–volume (*P–V*) curves from four different compression ramps on Ce and Pr are shown in Fig. 3, and those from the remaining ramps are shown in the Supplementary Material. For each material, both the *P–V* curves and the volume drop across the VC transition obtained at different compression rates are in good agreement with each other and also with results from previous static compression studies^20,21^. For both elements, the large overall volume changes experienced during the entire compression ramp (e.g. the volume of Pr decreases by 50% by ~ 40 GPa) raises the question of whether the fastest compression rates could result in a non-negligible increase in the sample temperature due to the short timescale of the experiment. In particular, accurate knowledge of the *P–T* path is important to rule out the effect of temperature on the phase transition pressure, which would shift the VC transition to higher pressures due to the positive *P*–*T* slope of the phase boundary.

In order to assess the possibility of sample heating during the rapid compression experiments on Ce, our dDAC results were compared to the 293 K and 377 K isotherms reported in the static compression study by Lipp et al.^21^ (Fig. 3). This allows us to look for deviations from the 293 K isotherm due to thermal expansion of the crystal lattice. It can be seen that both the γ- and α-Ce volumes plot below the 377 K data and are in good agreement with the data collected at 293 K, suggesting that there is no significant heating of Ce up to the maximum compression rate achieved in this study (472 GPa/s). However, we note that evaluation of heating above ~ 1.5 GPa is complicated by the fact that the 293 and 377 K *P–V* curves overlap in this region. A fractional volume drop of Δ*V*/*V* = 15.0 ± 0.4% was found for all the experiments, and no systematic changes as a function of compression rate are observed. Considering that the γ- to α-Ce phase boundary is very sensitive to temperature it is unlikely that there is significant heating of the sample during these runs since the first appearance of α-Ce is consistently observed between 0.70 and  0.77 GPa independent of the rate of compression.

Our Pr dDAC results were also compared to the 300 K and 540 K static compression isotherms reported by Baer et al.^20^ to assess possible heating of the sample. Unfortunately, the volumes obtained for the *d*-*fcc* phase are scattered, which makes it more difficult to identify temperature-induced deviations from the 300 K isotherm. This scatter is likely due to uncertainties in the fitted peak positions because of both peak overlap and low intensity of the diffraction peaks at short exposure times. A more detailed discussion on this topic can be found in the Supplementary Material. The *c/a* ratio of the *d*-*fcc* phase ranges from 2.40 to 2.52 (see Supplementary Material), in good agreement with previous results^20,31,36^. Most of the data points lie close to √6 = 2.449, which corresponds to the undistorted *fcc* structure, suggesting that the deviatoric stress in these Ne loaded dDAC experiments remains low. Most of the data points plot below the 540 K isotherm. Baer et al.^20^ reported a linear decrease in the fractional volume drop at the VC transition with temperature (Δ*V*/*V* = 16.235 − 0.0156[*T*(K)]), where this gradual decrease is in marked contrast to Ce. The average Δ*V*/*V* in our experiments is 10.2 ± 0.4%. If one takes the smallest value for Δ*V*/*V* of 9.7% it provides an upper bound of possible temperature increase of the sample of 120 K. However, as this approach relies on accurate atomic volumes of the un-collapsed and collapsed phases and given the scatter observed for the Pr *d*-*fcc* phase, this is not the most reliable approach for estimating temperature changes in these dDAC experiments.

**Figure 4:**
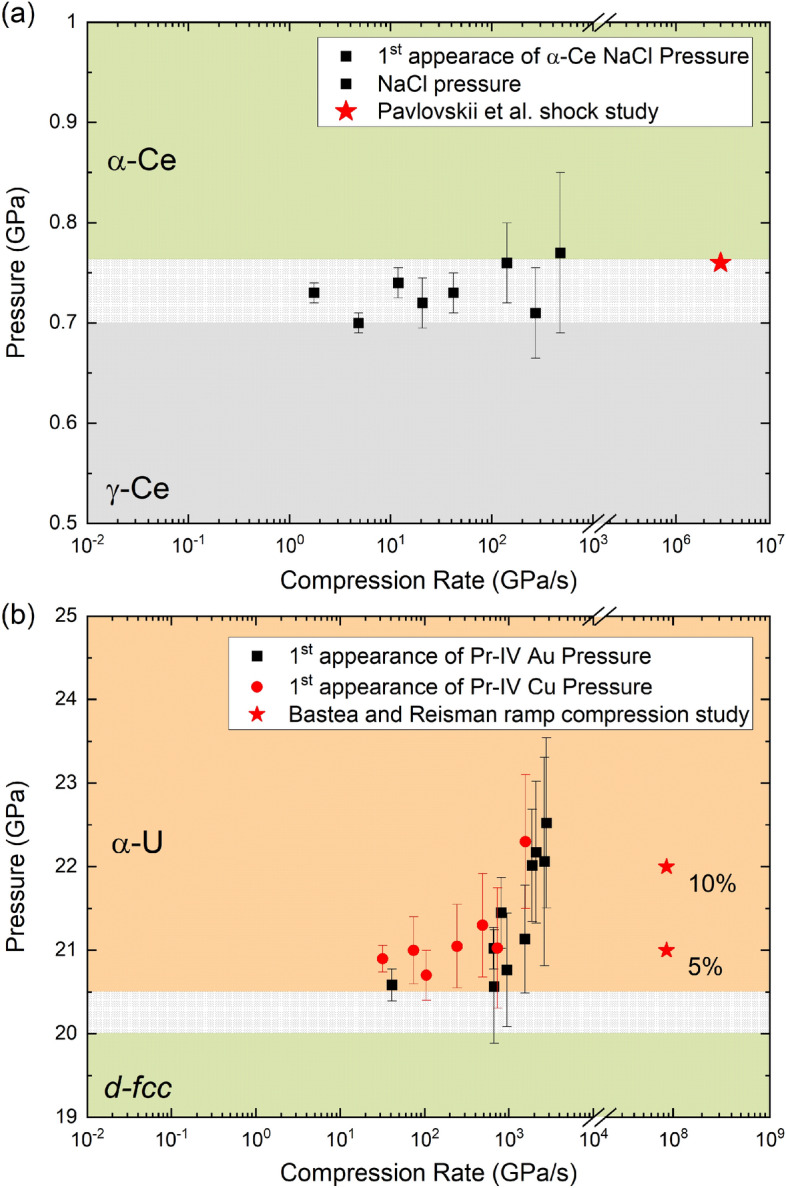
VC transition pressure as a function of compression rate for (**a**) Ce and (**b**) Pr. The dotted region is the reported range of the transition pressure as determined from static compression experiments found in the literature^20–22,25–27,30,31^ (where the compression rates are on the order of 0.01 GPa/s or less). These new dDAC results span nearly 4 orders of magnitude in compression rate and provide a clear picture of how Ce and Pr behave at intermediate timescales.

Figure 4 shows the compression-rate dependence of the onset transition pressure in Ce and Pr. The VC transition in Ce initiates at the same pressure regardless of compression rate, which is in contrast with Pr where the VC transition pressure *increases* with compression rate (i.e. an overdriving of the phase transition boundary). These new dDAC results span nearly 4 orders of magnitude in compression rate, bridging the gap between traditional “static” DAC experiments (time scales of minutes to hours) and dynamic shock and ramp compression experiments (µs–ns time scales). These results are consistent with the shock compression studies of Pavlovskii et al.^34^ who report that the VC transition in Ce occurs at 0.76 GPa, and also with ramp compression experiments on Pr by Bastea and Reisman^35^, who report a 5–10% over-compression at both the PrI-PrII(III) and the PrII(III)-PrIV phase transformations. As discussed above, this observation is not likely related to significant heating of the sample under rapid compression in the dDAC.

For Ce, the results from our dDAC experiments and those from previous shock compression studies^34^ both show that the VC transition pressure is independent of compression rate, indicating that the kinetics of the VC transition are fast in comparison to the compression timescales. These results are in agreement with previous work which found that cycling back and forth across the phase boundary at room temperature had no effect on the kinetics of the transformation^37^. A recent study interpreted the observation of two co-existing single crystals with the same crystal structure and orientation as evidence that the γ–α transition is a diffusionless phase transformation where nucleation and growth occurs at multiple sites^22^. This type of transformation is characterized by a small (usually less than interatomic distances) cooperative movement of a large number of atoms, which results in a change in crystal structure while the atoms maintain their relative positions. These are sometimes referred to as *military* transformations in contrast to *civilian* diffusion-based phase transformations^38^.

These results can be understood by considering the crystal structures of the γ and α phases of Ce. The γ to α transformation is isostructural so Ce remains *fcc* across the VC transition. Since there is no rearrangement of the atoms across the phase transition, this transition likely occurs more rapidly than a transition where rearrangement of the atoms is required. As for Pr, the VC transition is a structural transition from a higher symmetry phase (*d*-*fcc, R*$\overline{3 }$*m*) to a lower symmetry phase (α-U, *Cmcm*) requiring a rearrangement of the atoms. There is likely a much larger activation barrier to the atomic rearrangement concerning the VC of Pr versus Ce. Additionally, there may also be a change in multiplicity of the valence band across the VC transition in Pr^20^. Changes in multiplicity can be slow processes so it is possible that this may contribute to the overdrive of this transition with fast compression. Interestingly, there is also likely a change in multiplicity in Ce across the VC transition^39^ yet we do not observe an overdrive of the VC transition. Therefore, Ce may be unusual since it is an isostructural transition. It should be noted that there are two main models that have been proposed to explain the VC transition in Ce. The Mott transition model proposes that the nature of the 4f states changes from strongly correlated (localized) at pressures below the transition to more weakly correlated (itinerant) at pressures above the transition^40–42^, while the Kondo volume collapse model proposes that the conduction electron screening of the Ce 4f electron changes while the 4f electrons remain localized in both γ and α Ce^18,19^.

Figure 4 shows data from samples which were compressed multiple times in order to collect data points at different compression rates. Therefore, in order to definitively identify rate-dependent phenomena, it is necessary to rule out the possibility that the VC transition pressure is influenced by defect generation associated with the large volume change during the transition. For example, dislocation formation in Ce is a natural consequence of the lattice mismatch at the γ-α interface^23^. It has been proposed that each time the γ ↔ α transformation occurs within a compression cycle, more dislocations remain and deformation bands form near the (111) planes which could retard the transformation^23^. However, no evidence of any defect-generation induced raising or lowering of the γ-α phase transition pressure was observed in our experiments. The VC transition pressure was not observed to change in one of our Ce samples which was cycled through the transition a total of nine times, suggesting that more than nine cycles may be needed for defect generation to become relevant^18^. In Pr, defect generation was investigated by performing oscillation experiments similar to^43^, where the sample was subjected to 20 compression cycles at an average compression rate of 17 GPa/s. In this case, the Pr-III to Pr-IV phase boundary was crossed multiple times in a single experiment, and the transition pressure was observed to remain essentially constant as a function of compression cycle (Supplementary Material). This likely indicates that for Pr defect generation does not play a major role in the results shown in Fig. 4, and that possibly more cycles are needed before defect generation would play a role. This strongly suggests that the observed shift in transition pressure is purely related to the rate of compression i.e. the observation of kinetic phenomena.

## Conclusions

The VC transitions in Ce and Pr have been investigated across an unprecedented range of compression rates in the latest generation of piezo-driven dDAC. These new results span nearly 4 orders of magnitude in compression rate, bridging the gap between traditional “static” DAC experiments and dynamic shock and ramp compression experiments. The onset transition pressure of the VC transitions in Ce and Pr at compression rates up to the ~ 1000 GPa/s regime were accurately constrained. These results show that the kinetics of the γ- to α-Ce VC transition are fast, which is consistent with the shock compression study of Pavlovskii et al.^34^ and the cycling experiments reported by Kutsar^37^. Our results are also consistent with the proposed dislocation induced diffusionless growth mechanism proposed by Decremps et al.^22^, but they do not allow us to distinguish between the proposed Mott transition or Kondo screening models. In contrast, the onset transition pressure of the Pr-III to Pr-IV VC transition *increases* as the compression rate *increases*, in agreement with results from the ramp compression study of Bastea and Reisman^35^. Similar behavior was observed in Bi, where the Bi-III to Bi-V transition is observed at higher pressures at faster compression rates^16^. This may be characteristic of structural transitions where atomic rearrangements occur.

## Methods

Two Ce samples were prepared and loaded between two pre-compressed NaCl pellets which served both as the pressure medium and pressure marker. Six Pr samples were prepared and loaded with either Au or Cu pressure markers and a Ne pressure medium. Pressure markers NaCl, Au, and Cu were obtained from Alfa-Aesar all with a minimum purity 99.9%. The Ce and Pr samples were obtained from Alfa-Aesar as foil samples packaged in mylar under Ar. The Ce foil was 25 × 25 mm and 1 mm thick with a purity of 99.9% (Lot # B07X002), where as the Pr foil had the same dimensions and 99.5% purity (Lot # R19A044). Based on the peak widths of the measured ambient samples the grain size is estimated to be ~ 2–4 um. Since Pr and Ce rapidly oxidize in air, they were loaded in a dry N_2_ box and screened with X-rays prior to the dDAC experiments to ensure the samples were oxide free or nearly oxide free. DACs were equipped with standard design diamond anvils with culets ranging from 200 to 750 μm, and stainless-steel gaskets were used in all the experiments.

Time-resolved synchrotron X-ray diffraction experiments were performed on dynamically-compressed Ce and Pr samples at the Extreme Conditions Beamline (ECB, P02.2) at PETRA III^44^ using the LLNL dDAC design^14^. The experimental setup was described in greater detail by Husband et al.^16^. Data were collected using a 25.6 keV (~ 0.4843 Å) incident X-ray beam focused to a ~ 8(h) × 3(v) μm^2^ spot using compound refractive lenses. This energy was chosen because of the high flux that is achieved in this beamline configuration and the quantum efficiency of the GaAs Lambda detectors was almost 100%. Diffraction images were collected using two GaAs LAMBDA 2 M detectors that were horizontally offset to either side of the primary X-ray beam at a sample-to-detector (SDD) distance of ~ 420 mm. The SDD, detector tilt and rotation were calibrated based on a Cr_2_O_3_ NIST diffraction standard. The integration of the dDAC into the set-up at the ECB, including information on triggering signals for each component, is fully described in reference^14^. Samples were compressed using a trapezoidal voltage waveform with a variable rise time, where samples were compressed multiple times through the transition to collect multiple data points.

Diffraction images were radially integrated using DIOPTAS software^45^, which can perform bulk integration of the diffraction images for each compression cycle^45^. The integrated diffraction patterns were processed in OriginPro using a custom code written to process the large number of diffraction patterns collected for each dDAC run. Diffraction peaks were fit using a Pseudo-Voigt function and the refined peak positions were used to calculate the atomic volumes of the sample and pressure marker. The pressure during each compression-decompression cycle was estimated from the fitted Au (111), Cu (111), or NaCl (220) reflections using the reported EOSs for Au^46^, Cu^47^, and NaCl^48^ (see Supplementary Material for a more detailed description of volume calculations and tables of the pressure and volume from each run). The transition pressure for all these experiments is defined as the first appearance of α-Ce or Pr-IV in the diffraction patterns.

## Supplementary Information

Supplementary Information 1.

Supplementary Information 2.

## Data Availability

All data required to evaluate the conclusions of the manuscript are presented in the main text and/or the Supplementary Materials. Additional data related to this paper is available upon reasonable request from the corresponding author.

## References

[1] Holmes NC, Moriarty JA, Gathers GR, Nellis WJ (1989). The equation of state of platinum to 660 GPa (6.6 Mbar). J. Appl. Phys..

[2] Duffy TS, Smith RF (2019). Ultra-high pressure dynamic compression of geological materials. Front. Earth Sci..

[3] Lyzenga GA, Ahrens TJ (1979). Multiwavelength optical pyrometer for shock compression experiments. Rev. Sci. Instrum..

[4] Radousky HB, Mitchell AC (1989). A fast UV/visible pyrometer for shock temperature measurements to 20 000 K. Rev. Sci. Instrum..

[5] Grover R, Urtiew PA (1974). Thermal relaxation at interfaces following shock compression. J. Appl. Phys..

[6] Brantley DA, Crum RS, Akin MC (2021). Comparing temperature convergence of shocked thin films of tin and iron to a bulk temperature source. J. Appl. Phys..

[7] Martin LP, Patterson JR, Orlikowski D, Nguyen JH (2007). Application of tape-cast graded impedance impactors for light-gas gun experiments. J. Appl. Phys..

[8] Hall CA (2000). Isentropic compression experiments on the Sandia Z accelerator. Phys. Plasmas.

[9] Fratanduono DE, Smith RF, Braun DG, Patterson JR, Kraus RG, Perry TS, Arsenlis A, Collins GW, Eggert JH (2015). The effect of nearly steady shock waves in ramp compression experiments. J. Appl. Phys..

[10] Davis JP, Brown JL, Knudson MD, Lemke RW (2014). Analysis of shockless dynamic compression data on solids to multi-megabar pressures: Application to tantalum. J. Appl. Phys..

[11] Fratanduono DE (2021). Establishing gold and platinum standards to 1 terapascal using shockless compression. Science.

[12] Smith RF (2013). Time-dependence of the alpha to epsilon phase transformation in iron. J. Appl. Phys..

[13] Evans WJ, Yoo CS, Lee GW, Cynn H, Lipp MJ, Visbeck K (2007). Dynamic diamond anvil cell (dDAC): A novel device for studying the dynamic-pressure properties of materials. Rev. Sci. Instrum..

[14] Jenei Z (2019). New dynamic diamond anvil cells for tera-pascal per second fast compression x-ray diffraction experiments. Rev. Sci. Instrum..

[15] Gomez-Perez N, Rodriguez JF, McWilliams RS (2017). Finite element modeling of melting and fluid flow in the laser-heated diamond-anvil cell. J. Appl. Phys..

[16] Husband RJ, O'Bannon EF, Liermann HP, Lipp MJ, Mendez ASJ, Konopkova Z, McBride EE, Evans WJ, Jenei Z (2021). Compression-rate dependence of pressure-induced phase transitions in Bi. Sci. Rep..

[17] Johansson B (1974). The α-γ transition in cerium is a Mott transition. Philos. Mag..

[18] Allen JW, Martin RM (1982). Kondo volume collapse and the γ→α transition in cerium. Phys. Rev. Lett..

[19] Lavagna M, Lacroix C, Cyrot M (1982). Volume collapse in the Kondo lattice. Phys. Lett. A.

[20] Baer BJ, Cynn H, Iota V, Yoo CS, Shen GY (2003). Phase diagram and equation of state of praseodymium at high pressures and temperatures. Phys. Rev. B.

[21] Lipp MJ, Jackson D, Cynn H, Aracne C, Evans WJ, McMahan AK (2008). Thermal signatures of the Kondo volume collapse in cerium. Phys. Rev. Lett..

[22] Decremps F (2011). Diffusionless γ⇄α phase transition in polycrystalline and single-crystal cerium. Phys. Rev. Lett..

[23] Moore KT, Belhadi L, Decremps F, Farber DL, Bradley JA, Occelli F, Gauthier M, Polian A, Aracne-Ruddle CM (2011). Watching a metal collapse: Examining cerium’s γ↔α transformation using X-ray diffraction of compressed single and polycrystals. Acta Mater..

[24] Bradley JA (2012). 4f electron delocalization and volume collapse in praseodymium metal. Phys. Rev. B.

[25] O'Bannon EF, Pardo OS, Soderlind P, Sneed D, Lipp MJ, Park C, Jenei Z (2022). Systematic structural study in praseodymium compressed in a neon pressure medium up to 185 GPa. Phys. Rev. B.

[26] Lipp MJ (2012). X-ray emission spectroscopy of cerium across the γ-α volume collapse transition. Phys. Rev. Lett..

[27] Olsen JS, Gerward L, Benedict U, Itie JP (1985). The crystal structure and the equation of state of cerium metal in the pressure range 0–46 Gpa. Physica B.

[28] Vohra YK, Beaver SL, Akella J, Ruddle CA, Weir ST (1999). Ultrapressure equation of state of cerium metal to 208 GPa. J. Appl. Phys..

[29] Bridgman PW (1927). The compressibility and pressure coefficient of resistance of ten elements. Proc. Am. Acad. Arts Sci..

[30] Cunningham NC, Velisavljevic N, Vohra YK (2005). Crystal grain growth at the α-uranium phase transformation in praseodymium. Phys. Rev. B.

[31] Evans SR, Loa I, Lundegaard LF, McMahon MI (2009). Phase transitions in praseodymium up to 23 GPa: An X-ray powder diffraction study. Phys. Rev. B.

[32] Velisavljevic N, Vohra YK (2004). Distortion of alpha-uranium structure in praseodymium metal to 311 GPA. High Press. Res..

[33] Jensen BJ, Cherne FJ (2012). Jet formation in cerium metal to examine material strength. J. Appl. Phys..

[34] Pavlovskii MN, Komissarov VV, Kutsar AR (1999). Isomorphic γ→α phase transition of cerium under shock compression. Combust. Explos. Shock Waves.

[35] Bastea M, Reisman DB (2007). Near-equilibrium polymorphic phase transformations in praseodymium under dynamic compression. Appl. Phys. Lett..

[36] Hamaya N, Sakamoto Y, Fujihisa H, Fujii Y, Takemura K, Kikegawa T, Shimomura O (1993). Crystal structure of the distorted FCC high-pressure phase of praseodymium. J. Phys.-Condens. Matter.

[37] Kutsar AR (1979). γ reversible α-transformation and volumetric anomalies in cerium under pressure. Dokl. Akad. Nauk SSSR.

[38] Porter DA, Easterling KE (1992). Phase Transformations in Metals and Alloys.

[39] Johansson B, Abrikosov IA, Alden M, Ruban AV, Skriver HL (1995). Calculated phase diagram for the γ⇌α transition in Ce. Phys. Rev. Lett..

[40] McMahan AK (2005). Combined local-density and dynamical mean field theory calculations for the compressed lanthanides Ce, Pr, and Nd. Phys. Rev. B.

[41] Benedict U (1993). Pressure-induced phase transitions in 5f and 4f metals and compounds. J. Alloys Compd..

[42] Holzapfel WB (1995). Structural systematics of 4f and 5f elements under pressure. J. Alloys Compd..

[43] Marquardt H, Buchen J, Mendez ASJ, Kurnosov A, Wendt M, Rothkirch A, Pennicard D, Liermann HP (2018). Elastic softening of (Mg0.8 Fe0.2)O ferropericlase across the iron spin crossover measured at seismic frequencies. Geophys. Res. Lett..

[44] Liermann HP (2015). The extreme conditions beamline P02.2 and the extreme conditions science infrastructure at PETRA III. J. Synchrotron Radiat..

[45] Prescher C, Prakapenka VB (2015). DIOPTAS: A program for reduction of two-dimensional X-ray diffraction data and data exploration. High Press. Res..

[46] Fei Y, Ricolleau A, Frank M, Mibe K, Shen G, Prakapenka V (2007). Toward an internally consistent pressure scale. Proc. Natl. Acad. Sci. U.S.A..

[47] Holzapfel WB, Hartwig M, Sievers W (2001). Equations of state for Cu, Ag, and Au for wide ranges in temperature and pressure up to 500 GPa and above. J. Phys. Chem. Ref. Data.

[48] Dorogokupets PI, Dewaele A (2007). Equations of state for Cu, Ag, and Au for wide ranges in temperature and pressure up to 500 GPa and above. High Press. Res..

