# Supplementary material for: Dynamic compression of Ce and Pr with millisecond time-resolved X-ray diffraction
Source: Sci Rep. 2022 Oct 14;12:17294. doi: 10.1038/s41598-022-22111-5 (PMC9568586; doi:10.1038/s41598-022-22111-5)
Supplement: Supplementary file 2 — Supplementary Information 2. [file 41598_2022_22111_MOESM2_ESM.docx]

**REVISION 1**

**Dynamic compression of Ce and Pr with millisecond time resolved X-ray diffraction**

Earl F. O’Bannon^1,^*, Rachel J. Husband^2^, Bruce J. Baer^1^, Magnus J. Lipp^1^, Hanns-Peter Liermann^2^, William J. Evans^1^, Zsolt. Jenei^1^

^1^Physics Division, Physical & Life Sciences Directorate, Lawrence Livermore National Laboratory, Livermore, CA, 94551, USA

^2^Deutsches Elektronen-Synchrotron DESY, Notkestraße 85, 22607 Hamburg, Germany

*obannon2@llnl.gov


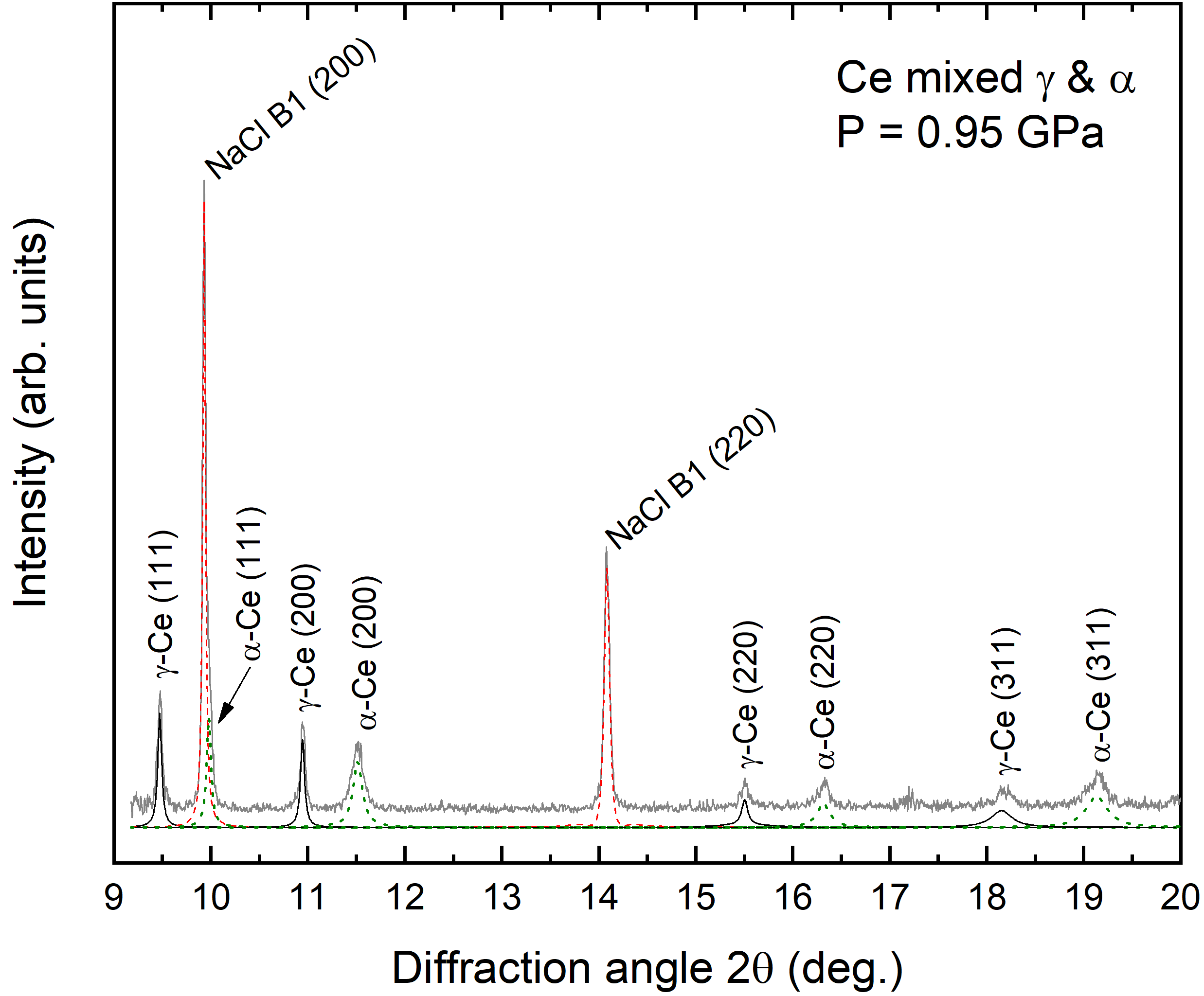


**Figure S1.** Mixed γ- and α-Ce diffraction pattern showing peakfit details for the sample and pressure marker. Pattern taken at room temperature and 0.95 GPa. The Ce volume was determined from Ce(200) reflection because the α-Ce(111) reflection overlaps with the NaCl-B1(200) reflection. For γ-Ce the volume determined from (111) is indistinguishable from the volume determined from (200).

**
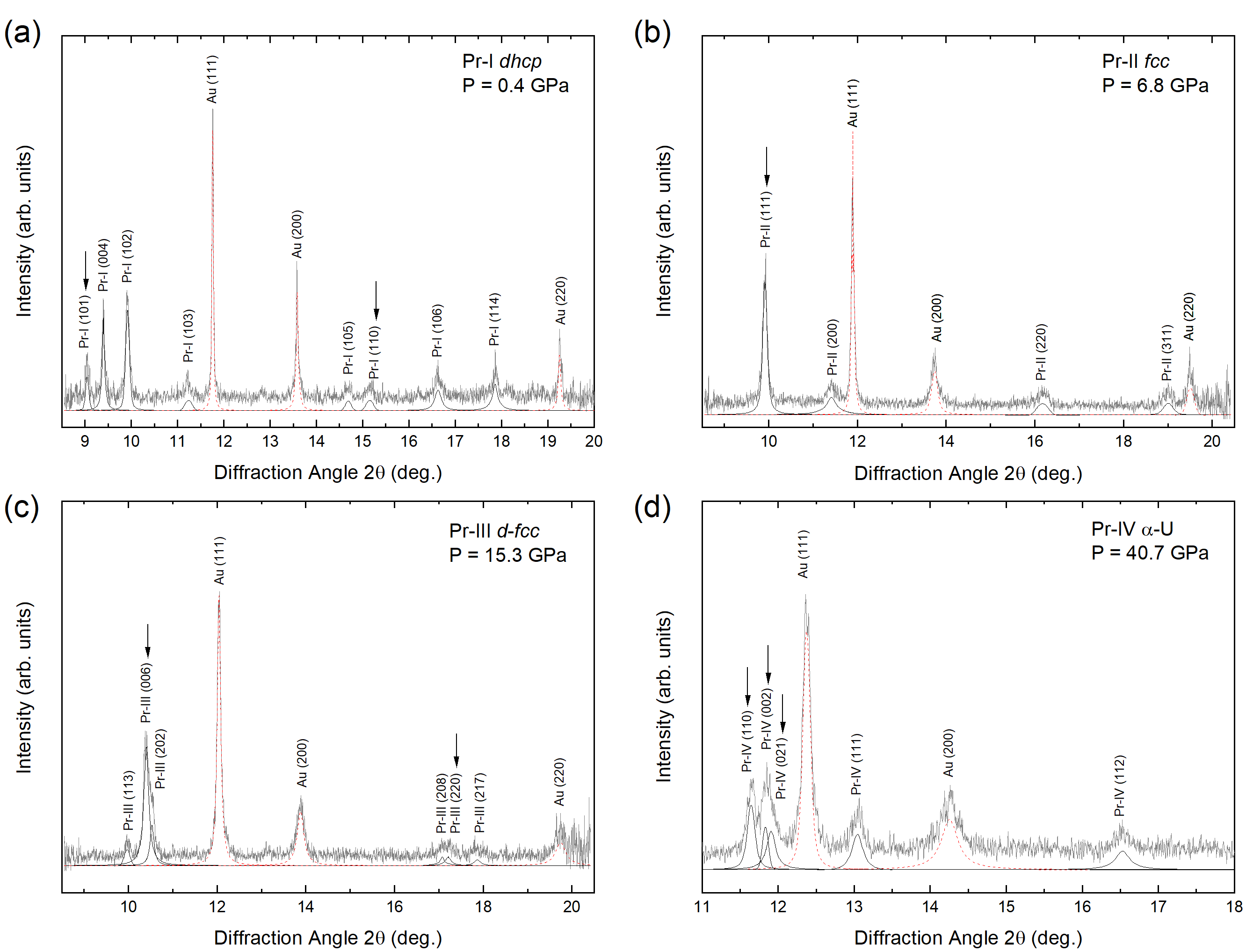
**

**Figure S2. (a)** Pr-I *dhcp* diffraction pattern taken at room temperature and 0.4 GPa showing peak fit, arrows indicate reflections used in volume calculations and red dashed lines are from the Au pressure marker. The *dhcp* volume calculated from (110) & (102) reflections, spacegroup *P*6_3_/*mmc*. **(b)** Pr-II *fcc* diffraction pattern taken at room temperature and 6.8 GPa showing peak fit, arrows indicate reflections used in volume calculations and red dashed lines are from the Au pressure marker. The *fcc* volume calculated from (111). **(c)** Pr-III *d*-*fcc* diffraction pattern taken at room temperature and 15.3 GPa showing peak fit, arrows indicate reflections used in volume calculations and red dashed lines are from the Au pressure marker. The *d-fcc* volume was calculated using the *hR*24 reported by Hamaya et al. (1993) spacegroup *R*$\bar{3}$*m* using the (006) and (220) reflections. The scatter observed in the obtained volumes is likely due to the low intensity of the Pr-III 208 and 220 reflections and the inability to reliably deconvolve two peaks in this region. In some cases, we fit the 208, 220 doublet with one peak and called this the 220 reflection since this reflection has a higher intensity than the 208. We also note that Evans et al. (2009) report a transition from *hR*24 to Pr-VII at 14 GPa and they discuss that the atomic volume data of the new phase which is stable at room temperature from 14-20 GPa lie on an extrapolation of the compressibility curve established by the *hR*24 data. Given the extremely short exposure times required for these fast compression experiments we are not able to resolve enough peaks to index the body centered orthorhombic (or slightly distorted) phase so we simply used the *hR*24 structure to calculate our volumes in the ~7-20 GPa pressure range. **(d)** Pr-IV α-U diffraction pattern taken at room temperature and 40.7 GPa showing peak fit, arrows indicate reflections used in volume calculations and red dashed lines are from the Au pressure marker. The α-U volume was calculated from the Pr-IV (110), (021), and (002) reflections, spacegroup *Cmcm*. Note that the (021) and (002) peaks are a doublet and not always well resolved in our diffraction patterns.


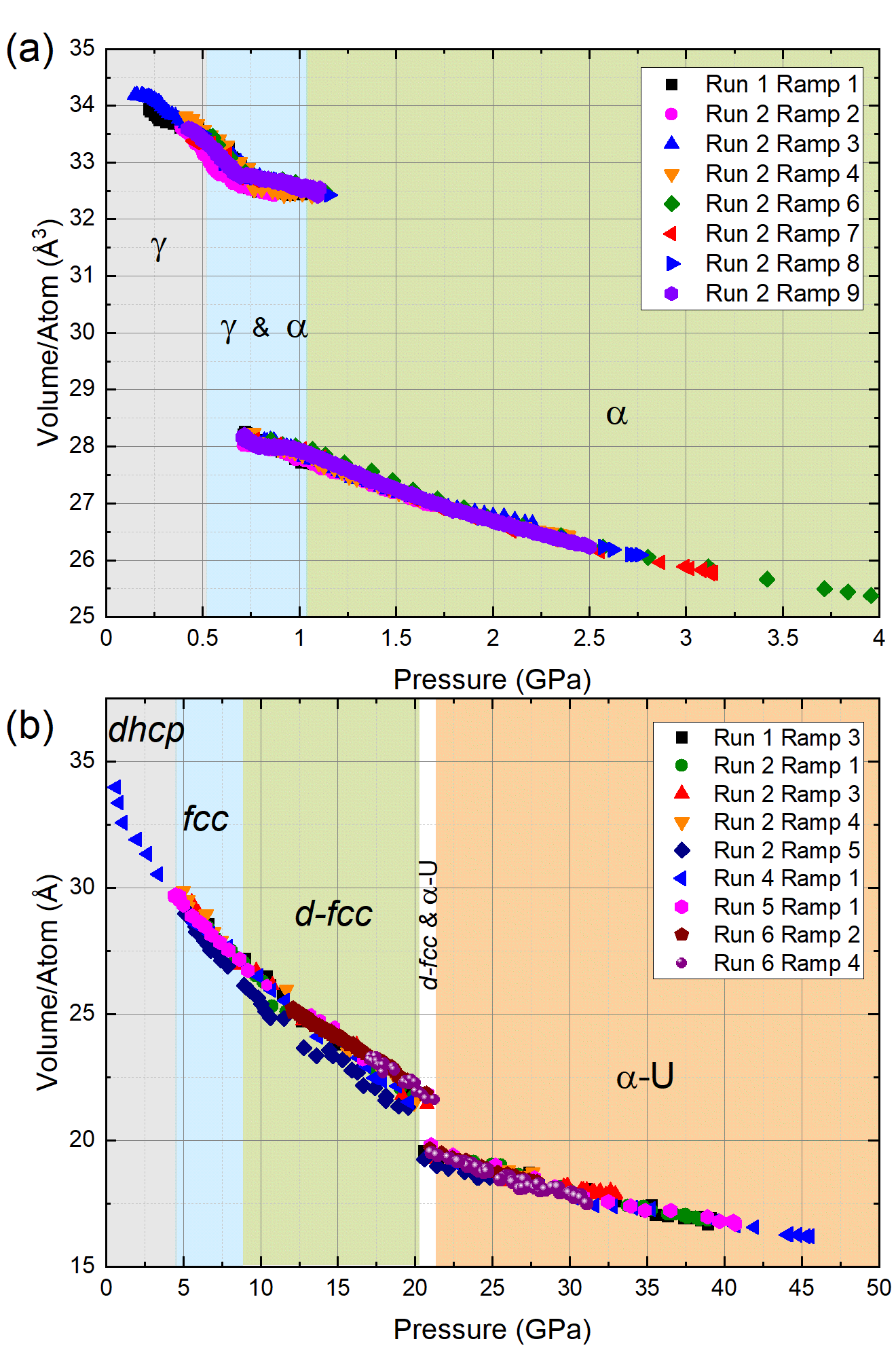


**Figure S3. (a)** Pressure volume curves from all Ce experiments. **(b)** Pressure volume curves from all Pr experiments where reliable volumes can be obtained for Pr-III.


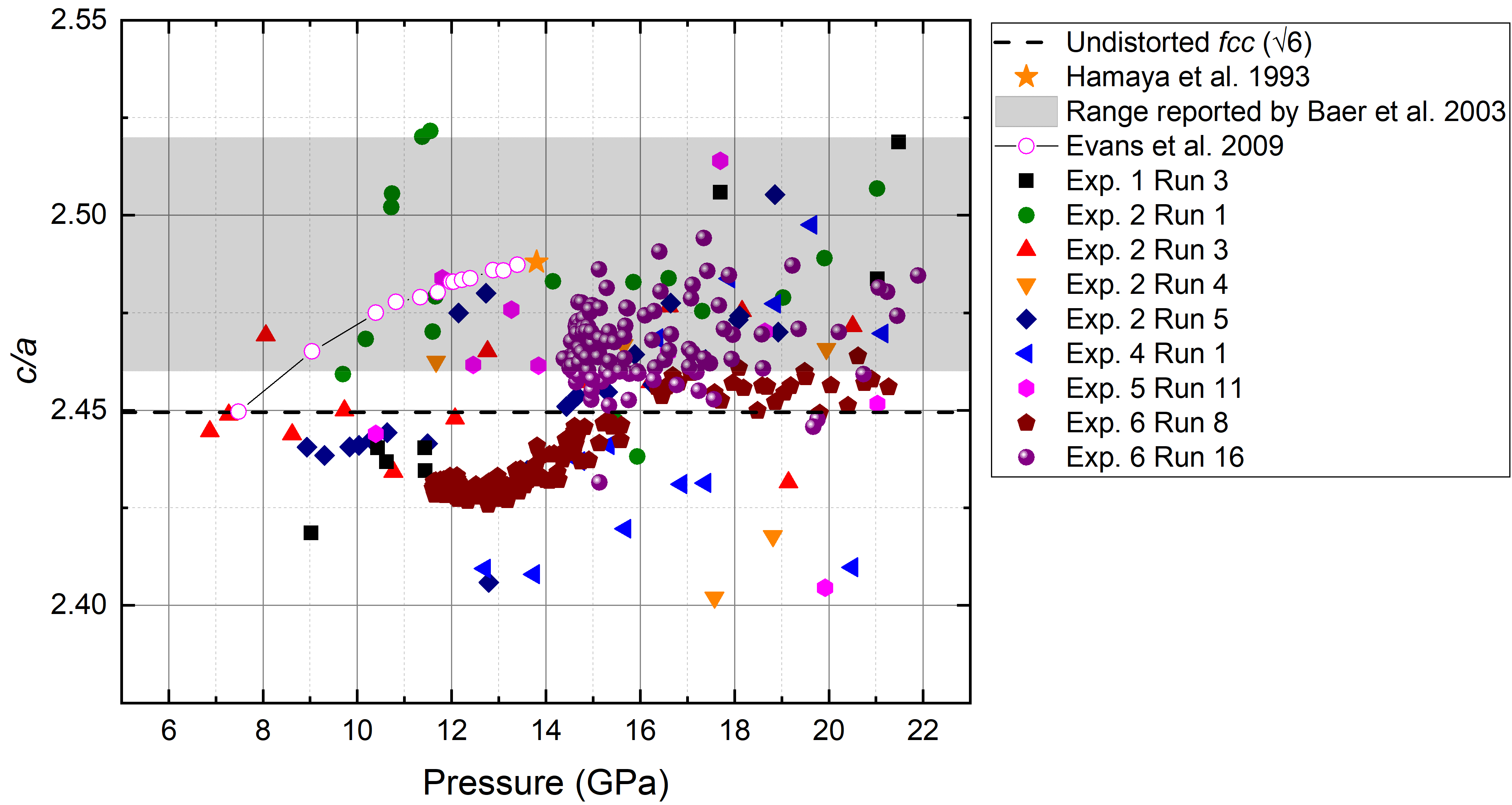


**Figure S4.** c/a ratio of *d*-*fcc* phase of Pr plotted as a function of pressure. The dashed line is at √6 which represents the undistorted *fcc* value. Previous results from Hamaya et al. (1993), Baer et al. (2003) and Evans et al. (2009) are also shown for comparison.


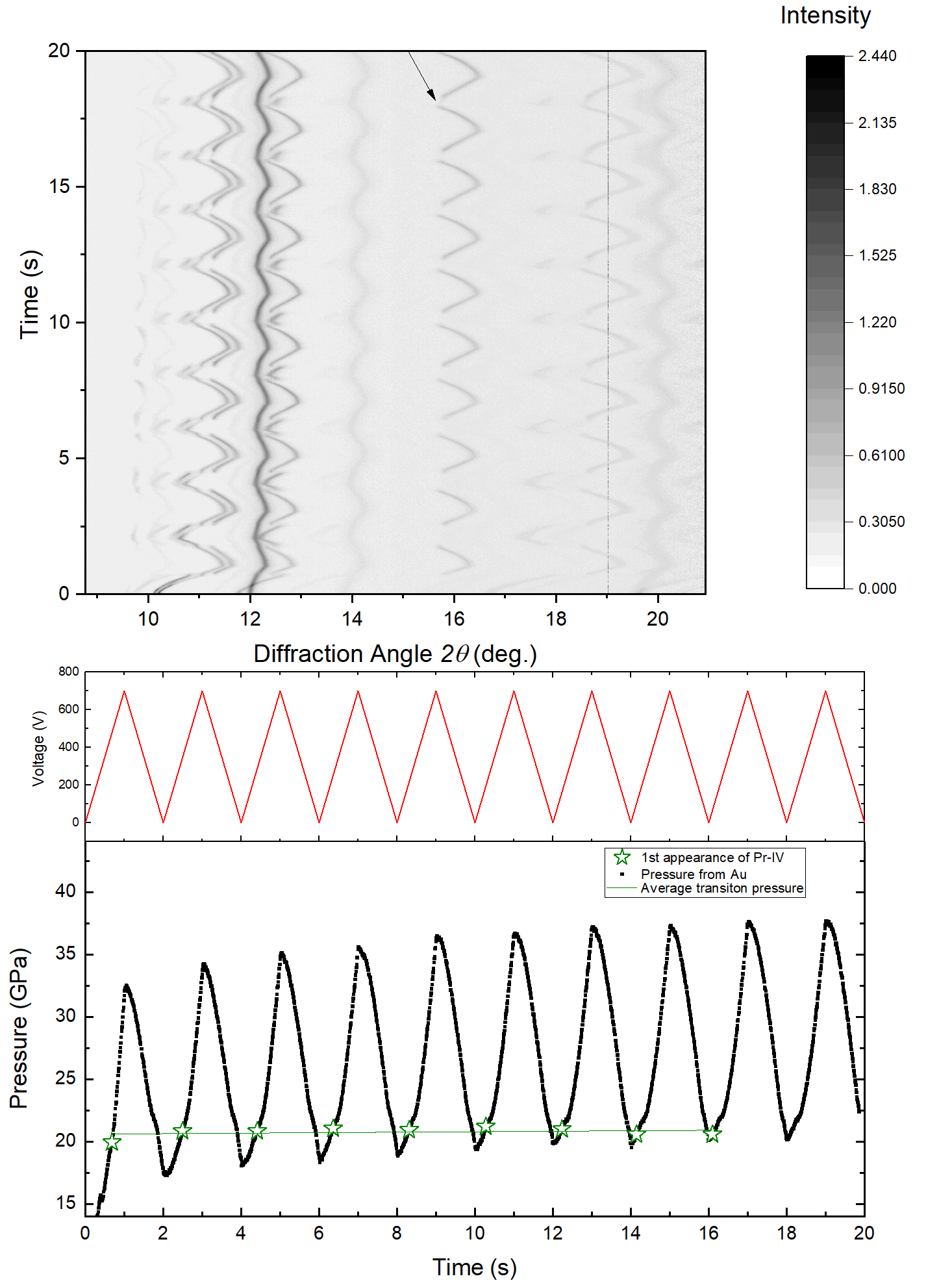


**Figure S5.** Upper panel is an intensity plot of the oscillation experiment from 4000 diffraction patterns the Pr-III to Pr-IV boundary was crossed multiple times in the same experiment. The arrow shows the Pr-IV (112) reflection (which was used to determine the onset pressure of the VC transition) after ~16 s this reflection no longer disappears since the pressure is not decreasing below ~20 GPa so Pr-III is no longer observed. In the lower panel is a time dependent pressure profile of Pr compressed through the Pr-III to Pr-IV transition nine times. The time dependent voltage applied to the piezo actuator consisting of a triangular waveform with a 1 s rise time. Lower panel is the pressure profile during the experiment, green stars indicate the 1^st^ appearance of Pr-IV which remains essentially constant through the experiment. After 16 seconds the pressure did not decrease below ~20 GPa so Pr-IV was not transforming back to Pr-III.

| **Exp. #** | **Sample** | **PTM** | **Pressure Marker** | **Run no.** | **Starting pressure (GPa)** | **Maximum pressure (GPa)** | **Rise Time (s)** | **Amplitude (V)** | **Average compression rate (GPa/s)** | **Acquisition time (ms)** | **Number of diffraction patterns per detector** | **VC transition pressure (GPa)** | **Volume change (%)** |
| --- | --- | --- | --- | --- | --- | --- | --- | --- | --- | --- | --- | --- | --- |
| 1 | Ce | NaCl | NaCl | 1 | 0.20 | 1.07 | 0.500 | 400 | 1.7 | 1.0 | 1700 | 0.73 (0.01) | 14.7 |
| 2 | Ce | NaCl | NaCl | 2 | 0.42 | 1.60 | 0.100 | 500 | 11.8 | 1.0 | 320 | 0.74 (0.02) | 15.0 |
|  |  |  |  | 3 | 0.15 | 2.20 | 0.100 | 700 | 20.5 | 1.0 | 310 | 0.72 (0.03) | 15.3 |
|  |  |  |  | 4 | 0.22 | 2.30 | 0.050 | 700 | 41.0 | 1.0 | 170 | 0.73 (0.02) | 14.1 |
|  |  |  |  | 6 | 0.55 | 4.06 | 0.025 | 700 | 140 | 1.0 | 95 | 0.76 (0.04) | 15.4 |
|  |  |  |  | 7 | 0.54 | 3.24 | 0.010 | 700 | 270 | 0.5 | 100 | 0.71 (0.05) | 15.0 |
|  |  |  |  | 8 | 0.46 | 2.82 | 0.005 | 700 | 472 | 0.5 | 70 | 0.77 (0.08) | 15.2 |
|  |  |  |  | 9 | 0.44 | 2.84 | 0.500 | 700 | 4.8 | 1.0 | 1520 | 0.70 (0.01) | 15.1 |

**Table S1.** Summary of Ce experimental parameters for all the ramps used in generating the main figures of the manuscript. Average compression rate is the maximum pressure minus the starting pressure divided by the time over which this pressure change occurred. Numbers in parenthesis for the transition pressure are the uncertainty.

*Exp. 2 Run 1 piezo not triggered*

*Exp. 2 Run 5 not ran because detector issues re-ran as run 6*

| **Exp. #** | **Sample** | **PTM** | **Pressure Marker** | **Run no.** | **Starting pressure (GPa)** | **Maximum pressure (GPa)** | **Rise Time (s)** | **Amplitude (V)** | **Average compression rate (GPa/s)** | **Acquisition time (ms)** | **Number of diffraction patterns per detector** | **VC transition pressure (GPa)** | **Volume change (%)** |
| --- | --- | --- | --- | --- | --- | --- | --- | --- | --- | --- | --- | --- | --- |
| 1 | Pr | Ne | Au | 3 | 6.7 | 39.9 | 0.05 | 800 | 665 | 1.0 | 200 | 20.56 (0.68) | 11.0 |
| 2 | Pr | Ne | Au | 1 | 6.0 | 38.7 | 0.05 | 500 | 656 | 1.0 | 200 | 21.04 (0.25) | 11.0 |
|  |  |  |  | 3 | 3.5 | 31.9 | 0.03 | 600 | 947 | 2.0 | 200 | 20.76 (0.68) | 10.7 |
|  |  |  |  | 4 | 3.7 | 28.0 | 0.03 | 700 | 809 | 2.0 | 200 | 21.45 (0.43) | 10.3 |
|  |  |  |  | 5 | 6.1 | 26.5 | 0.50 | 700 | 41.0 | 10 | 200 | 20.58 (0.19) | 10.1 |
| 3 | Pr | Ne | Au | 2 | 8.7 | 29.6 | 0.01 | 600 | 2085 | 0.5 | 140 | 22.17 (0.85) | * |
|  |  |  |  | 3 | 6.6 | 34.3 | 0.01 | 600 | 2770 | 0.5 | 140 | 22.53 (1.02) | * |
| 4 | Pr | Ne | Au | 1 | 0.5 | 46.8 | 0.03 | 600 | 1547 | 0.5 | 220 | 21.14 (0.64) | 9.8 |
|  |  |  |  | 2 | 16.1 | 34.9 | 0.01 | 500 | 1880 | 0.5 | 100 | 22.02 (0.67) | * |
|  |  |  |  | 3 | 16.1 | 29.2 | 0.005 | 500 | 2631 | 0.5 | 50 | 22.06 (1.25) | * |
| 5 | Pr | Ne | Cu | 1 | 4.8 | 41.5 | 0.05 | 500 | 734 | 1.0 | 250 | 21.03 (0.72) | 9.8 |
|  |  |  |  | 3 | 12.7 | 24.8 | 0.025 | 500 | 484 | 1.0 | 120 | 21.30 (0.62) | * |
|  |  |  |  | 6 | 11.6 | 50.8 | 0.025 | 800 | 1568 | 0.5 | 240 | 22.31 (0.80) | * |
| 6 | Pr | Ne | Cu | 2 | 11.7 | 27.6 | 0.50 | 500 | 32.0 | 2.0 | 800 | 20.94 (0.16) | 10.3 |
|  |  |  |  | 4 | 14.7 | 33.1 | 0.250 | 500 | 74.0 | 1.0 | 900 | 21.07 (0.30) | 9.9 |
|  |  |  |  | 6 | 15.0 | 25.5 | 0.100 | 700 | 105 | 1.0 | 400 | 20.72 (0.30) | * |
|  |  |  |  | 7 | 11.3 | 23.4 | 0.050 | 700 | 242 | 1.0 | 250 | 21.05 (0.50) | * |

**Table S2.** Summary of Pr experimental parameters for all the ramps used in generating the main figures of the manuscript. Average compression rate is the maximum pressure minus the starting pressure divided by the time over which this pressure change occurred.

**Unable to resolve Pr-III 220 reflection so volumes before the VC transition cannot be obtained in these runs.*

*Exp. 1 Run 1 pressure not high enough to cross Pr-III to Pr-IV boundary*

*Exp. 1 Run 2 pressure not high enough to cross Pr-III to Pr-IV boundary*

*Exp. 2 Run 2 pressure not high enough to cross Pr-III to Pr-IV boundary*

*Exp. 3 Run 1 piezo not triggered*

*Exp. 5 runs 2, 4, and 5 used for timing purposes piezo was not triggered*

*Exp. 6 Run 1 pressure not high enough to cross Pr-III to Pr-IV boundary*

*Exp. 6. Run 3 Did not fully transform to Pr-IV*

*Exp. 6 Run 5 pressure not high enough to cross Pr-III to Pr-IV boundary*
